# An outlook on self-assessment of homework assignments in higher mathematics education

**DOI:** 10.1186/s40594-018-0146-z

**Published:** 2018-12-27

**Authors:** Sarah Beumann, Sven-Ake Wegner

**Affiliations:** 1 School of Mathematics and Natural Sciences, University of Wuppertal, Gaußstraße 20, Wuppertal, 42119 Germany; 2 School of Science, Engineering & Design, Teesside University, Southfield Road, Middlesbrough, TS1 3BX UK

**Keywords:** Self-assessment, Self-evaluation, Self-regulation, Mathematics education, Tertiary education

## Abstract

**Background:**

We discuss first experiences with a new variant of self-assessment in higher mathematics education. In our setting, the students of the course have to mark a part of their homework assignments themselves and they receive the corresponding credit without that any later changes are carried out by the teacher. In this way, we seek to correct the imbalance between student-centered learning arrangements and assessment concepts that keep the privilege to grade (or mark) completely with the teacher.

**Results:**

We present results in the form of student feedback from a course on functional analysis for third- and fourth-year students. Moreover, we analyze marking results from two courses on real analysis. Here, we compare tasks marked by the teacher and tasks marked by the students.

**Conclusions:**

Our experiments indicate that students can benefit from self-assessment tasks. The success depends, however, on many different factors. Promising for self-assessment seem to be small learning groups and tasks in which a priori weaker students can catch up with stronger students by increasing their practising time.

## Self-assessment

In recent years, the possibilities to access mathematical knowledge have increased significantly due to the digitalization of classical media like textbooks, exercises, or model solutions and due to concepts such as blogs, internet forums, and online-available video-taped lectures. Modern teaching methods aim to facilitate the latter to improve students’ learning success. They achieve this by using student-centered learning arrangements such as problem-based learning, research-based learning, or other methods that give the students more freedom, but also assign more responsibility to them for their own learning outcome. However, when it comes to an assessment, often classical instruments, like graded homework assignments, weekly quizzes, or closed-book exams, prevail. The philosophy behind this paper is the idea of improving the imbalance between learning arrangements and assessment by sharing, to some extent, the teachers’ privilege to grade (or to mark) with the students. Our concrete aim is to strengthen the students’ sense of being responsible for their own learning process by sharing with them the control. This in turn encourages the students to employ the advantages of digitalization to increase their own learning success. In particular, they no longer feel the need to hide the sources of their ideas from the teacher, but can themselves evaluate their personal gain in knowledge, skills, and competencies that they have extracted from these sources. The latter is a very important aspect of modern student-centered education.

The idea of sharing the control over the learning process with the students is neither new nor a concept that can easily be realized in the classroom. Indeed, Klenovski’s (1995, p. 161) quotation from a 1994 interview with a college teacher has lost nothing of its relevance:

“Students have to learn that it’s their course, their learning and they have to take some control…it’s hard for some students because they want you to take control.”

However, from the mid-1990s on, different realizations of the idea have been surveyed in many areas of education such as chemistry (Davey 2015; Klenowski 1995), mathematics and statistics (De Corte et al. 1999; Olina and Sullivan 2004; Ross et al. 2001; 2002; Zuza et al. 2004; Stallings and Tascoine 1996), music (Hewitt 2011), and narrative writing (Ross et al. 1999) and with students of different ages and school types such as elementary school (Zuza et al. 2004), middle school (Hewitt 2011; Ross et al. 1999), and high school and college (Stallings and Tascoine 1996), to list only a sample. Some of these surveys mention a positive impact on the students’ achievement (Fernandes and Fontana 1994; Ross et al. 2002; Zuza et al. 2004; Stiggins and Chappuis 2005); some mention no impact (Hewitt 2011; Ross et al. 2001); some point out that self-assessment is not always precise (Basnet et al. 2012; Davey 2015). A positive influence on meta-competencies like self-efficacy (Ross et al. 2002), self-confidence (Olina and Sullivan 2004), active learning and motivation (Fernandes and Fontana 1994), and critical thinking and the ability to reflect on own work (Cooper 2006) is mentioned. In (De Corte et al. 1999) it is pointed out that appropriate beliefs about mathematics and mathematical learning are an important precondition.

In the papers cited above, rather different approaches are outlined about how to share control with the students in a concrete classroom situation. In this paper, we follow mostly the ideas of Klenovski (1995) who used the two notions of *self-evaluation* and *self-assessment*. Indeed, Klenovski (1995, p. 155–160) identifies “three key dimensions of the student self-evaluation process […]: the use of criteria by students to self-evaluate their own learning […]; the interactive dialogue […] between student and teacher, during the analysis of the student’s self-evaluation; [and] the ascription of a grade by the students for their own work.” Klenovski (1995, p. 147) states that “self-evaluation […] is broader than self-assessment in that the student is engaged in more than just deciding what grade he or she should get.” It appears to us that in the classroom situations surveyed by Klenovski the students did not have the final authority about the grade, but that the teacher could intervene (Klenowski 1995, second interview on p. 159), or an intervention by peer-learners was possible (Klenowski 1995, interview on p. 158). In our experiments, it is essential that the students ascribe their own grades (or marks) *without the intervention* of a second party. For this reason, we stick below to the word *self-assessment* although, of course, the use of criteria and a dialogue about assessment are important in our setting as well. Our incentive behind this concept of self-assessment—which differs from our knowledge of all concepts discussed so far in the literature—is the following:

1. Self-assessment allows us to give meta-tasks to the students that cannot be marked by the teacher. Examples could be to repeat some topic from the last year’s course or to practise a method “until the students master it.”
2. Self-assessment allows us to give extra tasks to the students, and to grant credit for working on these tasks, without the school having to pay staff that carries out the marking.
3. Self-assessment helps to illustrate that checking the validity of a proof is not a formal and fail-safe procedure but requires careful work and may depend on personal taste. This is for example the case when it comes to the amount of details that are given and the strategy that is pursued. In this sense, self-assessment generates appropriate beliefs about mathematics.
4. Self-assessment transfers to the students, for a moment, the full responsibility for their grading (or marking) and thus fosters the development of the earlier mentioned meta-competencies—like self-efficacy, self-confidence, and motivation—compared to situations in which students participate in the evaluation but the final grading (or marking) is done by the teacher.
5. Self-assessment encourages the students not just to maximize the teacher-assigned grade but to learn mathematics on a level of deep understanding.

Let us give two examples of authentic classroom situations that illustrate our incentive behind this article. In situation 1, a student kept asking for help with an exercise until the teacher solved the whole task for the student. As the solution is now of course correct, the teacher assigned, after it was handed in, the maximum number of marks. The student’s learning progress might however be poor as mathematics is not about applying internalized techniques to well-known problems, but about finding new techniques to solve unknown problems—which students only learn by solving problems on their own. In situation 2, the student hands in a solution copied from a book or from the internet. From the solution, the teacher can see that it was copied without any understanding, e.g., as it follows a naming convention different from the lecture, or as the notation is completely different from that on the problem sheet. As the math is however correct, the teacher feels that he cannot deduct much from the full score. The student’s learning progress is, however, more or less zero. Our initial idea was that giving the power and duty of marking to the students in such situations could result in a change of their beliefs. It could help the students to reconsider their strategies and become aware of their own responsibility—for their learning progress and for the mathematical work that they produce.

Let us mention that our basic idea of giving more control to the students in order to improve the learning process is also the leitmotif in Klenovski’s paper (Klenowski 1995). His findings (Klenowski 1995, p. 161f) support the latter statement but also point out that pedagogical change is needed and implementations of the concept have to be further studied. The first results explained below confirm that our new concept of intervention-free self-assessment can be applied successfully in higher mathematics education. On the other hand, they also identify drawbacks and obstructions. This paper is intended as a small preview and an invitation to other university teachers to contribute with their ideas and experience to the development of self-assessment in mathematics.

## A pilot study—first results on self-assessment

**Fig. 1:**
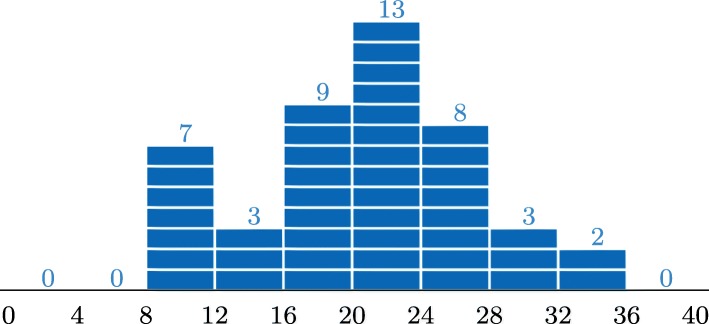
Teacher’s assessment: 0 students were awarded *n*∈[0,8] marks, 7 students were awarded *n*∈(8,12], etc

**Fig. 2:**
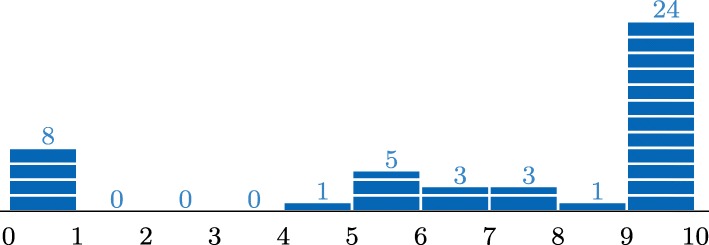
Self-assessment: 8 students awarded themselves *n*∈[0,1] marks, 0 students awarded themselves *n*∈(1,2], etc

In this section, we outline first experiences with our concept of self-assessment by presenting students’ feedback and the marks of two homework assignments. We compare the results of parts that were assessed by the teacher with parts that were assessed by the students (Figs. 1 and 2 and Tables 1 and 2). We present and discuss some selected feedback that gives insight into the students’ beliefs about their role in the learning and assessment process.

**Table 1:** Min = 0 and max = 40 for exercises marked by the teacher and min = 0 and max = 10 for the exercise marked by the students

	N	M	SD
Teacher’s assessment	45	21.09	6.73
Self-assessment	45	7.31	3.04

**Table 2:** Min = 0 and max = 90 for exercises marked by the teacher and min = 0 and max = 10 for the exercise marked by the students

	N	M	SD
Teacher’s assessment	7	56.86	16.00
Self-assessment	7	7.86	3.67

### Homework assignments in higher mathematics

The first experience of the authors with self-assessment was the spontaneous idea to assign the review of topics that had been covered in a previous course as a homework assignment. In order to underline that we wanted this to be understood as a serious task we decided to put it in the following form as one of four tasks on the weekly exercise sheet.

#### **Exercise 1**

(5 marks) Review the construction of the Lebesgue integral, the dominated convergence theorem and the monotone convergence theorem. Maybe it is helpful to browse the appendix of the book *(*Werner 2007*)* by D. Werner.

This task was given in the middle of a 14-week course on the foundations of functional analysis taught in 2012 with approximately 20 students in their third and fourth years. Each exercise sheet contained four tasks for which solutions had to be handed in and that were usually marked by the teacher. On this particular sheet, only three tasks required a solution. For the forth one, Exercise 1, the students were required to self-assess their achievement and to indicate the score on the submission. When we handed out the sheet, the students appeared very surprised and suspicious because they were not used to exercises of this type. Many of them did not award themselves the full amount of five marks. Indeed, they assumed that we would carry out some kind of “double checking,” like an oral examination during the recitation, if they assign themselves a high score. After the semester, we received the following feedback by one of the students.

“The exercise to recall the introduction (definition and main properties) of the Lebesgue integral and to give yourself marks on the basis of your comprehension is meaningful and helpful as well. First, one recalls the content carefully which leads to a deep understanding, and second the already rehearsed content anchors in memory. Since one gives marks on the basis of comprehension, you repeat the content carefully to ‘obtain’ a good score. Indeed, in order to avoid an embarrassing situation where the tutor checks that the number of marks is inappropriate, you think twice of how many marks are eligible.”

We mention that Exercise 1, as stated above, was the only self-assessed assignment in this course. The five marks correspond to approximately 2% of the total score of 260 marks that the students could achieve on the 13 exercise sheets.

Our second experience with self-assessment was the following. During a 14-week course on real analysis, taught in 2013 for first-year students, we gave the following two exercises. Both were given as additional exercises and were credited with 20 marks. The total of regular marks was 480. The self-assessment homework thus counted as approximately 4% extra credit.

#### **Exercise 2**

(10 marks) Become confident with handling sequences and with computing their limits, e.g., by working on the exercises from the additional worksheet on the course’s website.

The additional worksheet contained 46 sequences for which the limits had to be computed. The second exercise refers to the following theorem that establishes some basic rules for computations with convergent series.

#### **Theorem 1**

Let $(a_{k})_{k\geqslant 0}$, $(b_{k})_{k\geqslant 0}\subseteq \mathbb {R}$ and $\lambda \in \mathbb {R}$ be given.

- We have
  $${\sum\limits}_{k=0}^{\infty}a_{k}+\lambda{\sum\limits}_{k=0}^{\infty}b_{k} = {\sum\limits}_{k=0}^{\infty}a_{k}+\lambda b_{k} $$
  provided that the two series on the left are convergent.
- Assume that there exists $k_{0}\geqslant 0$ such that *a*_*k*_=*b*_*k*_ holds for all $k\geqslant k_{0}$. Then, the series over all *a*_*k*_’s converges if and only if the series over all *b*_*k*_’s converges.
- Let the series over the *a*_*k*_’s be convergent and let $(j_{k})_{k\geqslant 0}$ with *j*_*k*_↗*∞* and *j*_0_=−1 be given. Then, the following series
  $${\sum\limits}_{k=0}^{\infty}a_{j_{k}+1}+\cdots+a_{j_{k+1}} $$
  is also convergent. The converse is false.

During the lectures, we presented Theorem 1 without its proof. The exercise was then as follows.

#### **Exercise 3**

(10 marks) Make sure that you are able to prove the rules for computations with convergent series given in Theorem 1, e.g., by giving all or a suitable selection of the proofs yourself.

As in Exercise 1, we asked the students to award themselves the corresponding marks and to indicate the score on their submissions. They did neither get a model solution or a marking scheme. This reflects one of the main incentives for self-assessment mentioned in the beginning: Leaving the proofs completely to the students will grow their ability to evaluate if a mathematical argument is correct or not *by themselves*.

We mentioned that Exercise 2 appeared as an additional task on one of the homework sheets. On this sheet, four exercises were graded by the teacher and one exercise was subject to self-assessment. The following table shows the averages of the teacher-assessed part and the averages of the self-assessed part (Table 1). It is eye-catching that in this case the average of the teacher assessment is approximately 53% whereas the average of the self-assessment is approximately 75%.

The distribution of the teacher-assessed tasks (Fig. 1) looks Gaussian-like if one ignores the 13% of students that obtained less than or equal to 10 out of 40 marks. In this course, 50% of the marks on the sheets were sufficient to be admitted to the final exam. The grade for the course depended only on this exam. In view of this, the latter seems reasonable and expectable.

The distribution of the student-assessed tasks (Fig. 2) looks completely different and has a higher average. We prefer to be careful with drawing conclusions, since we compare exercises on different topics and with different levels of difficulty. It is, however, again eye-catching that 53% of the students awarded themselves the full 10 marks, whereas 18% awarded themselves zero marks.

It seems very interesting and important to us that among those eight students that assigned themselves zero marks, only one received 10 of 40 marks from the teacher. The other seven received between 17 and 26 out of 40 and thus scored around the average value. Among the 24 students that gave themselves the full 10 marks, we find five out of those six students that received less than or equal to 10 marks in the teacher-assessed part. This suggests that weak students in particular did not assess themselves very honestly. For a further development of self-assessment techniques, this effect has to be taken into account. More experiments are needed to see if the latter is a general trend or if the students in the long term will assess themselves in a reasonable fashion.

The third experiment on self-assessment was part of a 12-week course on real analysis for first-year students taught in 2018. We mention that we had a very small group of only seven students and thus an atmosphere in which the students know each other well and talk much about math, homework, exams, etc. The assessment consisted of a final exam and one longer homework assignment in the middle of the course. Both components contributed 50% to the final grade. The homework assignment consisted of 10 questions. It covered elementary logic, sets, mappings, and mathematical induction. One of the 10 questions was the following.

#### **Exercise 4**

(10 marks) Become confident with using truth tables by verifying a suitable sample the following statements:

1. *A*∧ T⇔*A*, *A*∨ F⇔*A*
2. *A*∨ T⇔ T, *A*∧ F⇔ F
3. *A*∨*A*⇔*A*, *A*∧*A*⇔*A*
4. ¬(¬*A*)⇔*A*
5. *A*∨*B*⇔*B*∨*A*, *A*∧*B*⇔*B*∧*A*
6. *A*∨(*B*∨*C*)⇔(*A*∨*B*)∨*C*
7. *A*∧(*B*∧*C*)⇔(*A*∧*B*)∧*C*
8. *A*∨(*B*∧*C*)⇔(*A*∨*B*)∧(*A*∨*C*)
9. *A*∧(*B*∨*C*)⇔(*A*∧*B*)∨(*A*∧*C*)
10. ¬(*A*∧*B*)⇔¬*A*∨¬*B*
11. ¬(*A*∨*B*)⇔¬*A*∧¬*B*
12. ¬(*A*∧*B*)⇔¬*A*∨¬*B*
13. ¬(*A*∨*B*)⇔¬*A*∧¬*B*
14. (*A*⇒*B*)⇔(¬*A*∨*B*)
15. *A*∨¬*A*, ¬(*A*∧¬*A*)
16. [(*A*⇒*B*)∧¬*B*]⇒¬*A*
17. [(*A*⇒*B*)∧(*B*⇒*C*)]⇒(*A*⇒*C*)
18. (*A*∧*B*)⇒*A*, (*A*∧*B*)⇒*B*
19. *A*⇒(*A*∨*B*), *B*⇒(*A*∨*B*)
20. (*A*⇔*B*)⇔[(*A*⇒*B*)∧(*B*⇒*A*)]
21. (*A*⇒*B*)⇔(¬*B*⇒¬*A*)
22. [(*A*∨*B*)∧¬*A*]⇒*B*
23. [(¬*A*∧*B*)⇒*F*]⇒(*A*⇒*B*)
24. [(*A*⇒*B*)∧*A*]⇒*B*

Indicate the number of marks on your submission. Don’t hand in any truth table!

Exercise 4 contributed 5% to the final mark. The design was similar to Exercise 2, where we gave 46 sequences to practise the computation of limits. However, we point out that the computation of these limits in most cases involved a certain trick, like applying an estimate, or combining two previous limits in a suitable way. In contrast to this, Exercise 4 was much more straightforward and can be completed—once the principle is understood—by a rather “mechanical procedure.”

In Table 2, we compare again the grading results of the self-assessed part with the teacher-assessed part. In our small group of seven students, the average of the tasks assessed by the teacher was with 63% lower than the 79% of the self-assessed part. This was also the case with Exercise 2. The correlation between the marks that the students gave themselves and the marks that the teacher gave to them was 0.77 in the current experiment. In the previous experiment, the correlation was only 0.05. One might conclude from this that the students’ evaluation of their own abilities in this case was closer to the teacher’s evaluation of the latter. However, we would like to be cautious here in view of the small group size and the different types of questions in Exercise 2 and Exercise 4. On the other hand, we are indeed convinced that this last experiment with self-assessment was more successful than the previous one. We recognized that some of the students put much effort into Exercise 4 and indeed did all 31 truth tables. By doing this, they gained not only the desired proficiency with the method. At the same time, they gained confidence in their own abilities and handed in their solutions with the good feeling that they really deserve the 10/10 marks that they ascribed to themselves. With a classical design (one or two of the statements listed in Exercise 4 to be handed in and to be marked by the teacher), we could not have achieved this.

### Students’ impressions about responsibility

The last experience that we want to discuss here did not involve self-assessment in the sense of our first section. It was, however, similar in the sense that the responsibility to work on homework assignments was completely due to the students. In contrast to the situations explained above, the marking was waived completely. In a third-year course with approximately 10 students and in a second-year course with approximately 50 students, we strongly recommended intensive work on the weekly assignments. We emphasized that the final exam will be very similar to the tasks in these assignments. In the small course, we asked the students to present their solutions during the exercise sessions. In the large course, the solutions were presented by the teacher and later uploaded to the website of the course, as there were too many participants for individual presentations. The grade for both courses was given on the basis of the final exam. During the term, we received much negative feedback. Indeed, most of the other teachers employed homework assessment, quizzes, midterm exams, and strict attendance requirements to control the students’ engagement. In view of the exam outcome, one can say that our concept completely failed in this context. In the middle of the course, we already recognized that only less than one quarter of the students downloaded the exercise sheets before the lesson. The whole situation is very well summarized by the following feedback comment.

“100% final is … strange … it has good and bad sides. Bad thing is that the students sometimes ‘forget’ about this course for the whole semester, which affects their final preparation.”

From this, one can deduce that the students were indeed aware that they did not assume responsibility for their own learning progress. However, it was us who did not manage to initiate a change of their learning behavior in this course. On the other hand, we received the following positive comment.

“Learning the subject WITHOUT WORRYING that you fail quiz or midterm and don’t have chance to pass the course. Learning with our own pace. Mock exams and homeworks help much. It seems risky and stressful at the end. But I think having too much midterms and quizzes gives constant stress which makes hard student life for low-pace studiers.”

This comment suggests that a paradigm shift might have been possible, but would had required a different methodology. Self-assessment—that we unfortunately did not use in this case—could have improved the situation.

## Discussion and outlook

Self-assessment in the sense of this article can be used successfully in higher mathematics education. The feedback from our third-year course on functional analysis indicated that students assessed themselves honestly or even too cautiously. In the first experiment with first year students, the data indicates that on average students overrated themselves within the self-assessed tasks and that in particular the very weak students did this excessively. Of course it is also possible that the teacher underrated certain students in the non self-assessed tasks. Indeed, it is a key problem of assessment that the latter is always subjective and individual. In view of the low weight (≤ 5*%*) of the self-assessment tasks, we consider the overrating as a tolerable side-effect. The setting of a small group and a task such as Exercise 4—in which everybody can achieve the full score by hard work—turned out to be very suitable for self-assessment. This setting in particular seems to grow the weaker students’ confidence in their own abilities. We point out that our concept differs substantially from previous implementations of self-evaluation due to the fact that students actually mark their own work without interventions of peers or the teacher. In particular, the first and the last feedback comment that we received suggest that this amplifies the belief that an effective learning process has to be designed by teachers and students together.

Our first explorative results also identify drawbacks and obstructions. The second last comment illustrates that it can be very difficult to achieve that students develop a sense of responsibility. In certain environments, it might even be impossible. Our experiments highlight that we cannot expect a priori that students will grade themselves honestly. Therefore, sophisticated implementations need to be designed in the future. In order to improve our concept, we aim to get an in-depth look into the self-evaluation process itself. It would be desirable to obtain more information on how the students actually ascribe the marks. However, collecting the students’ solutions and assessing their assessment—even if only for research purposes—might already influence the self-assessment. It seems to us that there is no easy or standard way to implement self-assessment.

To conclude, we like to mention once more that this small preview is intended as an invitation to other university teachers to contribute with their ideas and experience to the topic of self-assessment in mathematics. Larger experiments, which will follow the lines sketched above, are under preparation.

## Abbreviations

M: Mean value
N: Sample size
SD: Standard deviation
